# Phase‐Controlled Asymmetric Resonances for Enhanced Spectral Slope in a Ge‐OI Microring Resonator at 4.2 μm

**DOI:** 10.1002/nap2.70321

**Published:** 2026-10-14

**Authors:** Jinha Lim, SangHyeon Kim

**Affiliations:** ^1^ School of Electrical Engineering Korea Advanced Institute of Science and Technology (KAIST) Daejeon South Korea; ^2^ Center for Quantum Technology Korea Institute of Science and Technology (KIST) Seoul South Korea

## Abstract

Microring resonators (MRRs) are key components of photonic integrated circuits for filtering, modulation, and spectroscopic sensing. In the mid‐infrared (mid‐IR), asymmetric resonances are attractive because their steep local spectral transitions can enhance wavelength discrimination in compact resonant devices. Here, we demonstrate geometry‐controlled asymmetric resonances in a germanium‐on‐insulator (Ge‐OI) MRR at 4.2 μm by introducing a single subwavelength air hole into the bus‐waveguide coupling region as a localized phase perturbation. Effective‐index analysis shows that the hole geometry estimates a controllable phase shift Δ*ϕ* of up to 0.82π rad, enabling systematic evolution from a nearly symmetric Lorentzian resonance to a strongly asymmetric Fano‐like response. The results are explained by a phase‐modified transmission model and are described using a weighted Fano–Lorentz formalism, which explains the resonance evolution in the (*δ*, *η*) parameter space. Among our devices, the optimum point is achieved at an intermediate perturbation rather than at the maximum Δ*ϕ*, yielding a loaded quality factor *Q* of 2.15 × 10^5^ and a slope rate of 435 dB/nm. These results show that compact air‐hole perturbation can tune high‐*Q* asymmetric resonances in Ge‐OI mid‐IR photonics, where the useful high‐slope regime is determined by a balance among phase‐induced asymmetry, linewidth preservation, coupling condition, and local transmission contrast.

## Graphical abstract

A localized air‐hole perturbation in a mid‐infrared Ge‐on‐insulator microring enables phase‐controlled evolution from Lorentzian‐like to Fano‐like resonances. The strongest spectral discrimination emerges at an intermediate perturbation, where a loaded *Q* of 2.15 × 10^5^ and a slope rate of 435 dB/nm are achieved, revealing a practical route to high‐*Q*, high‐slope mid‐IR photonics.

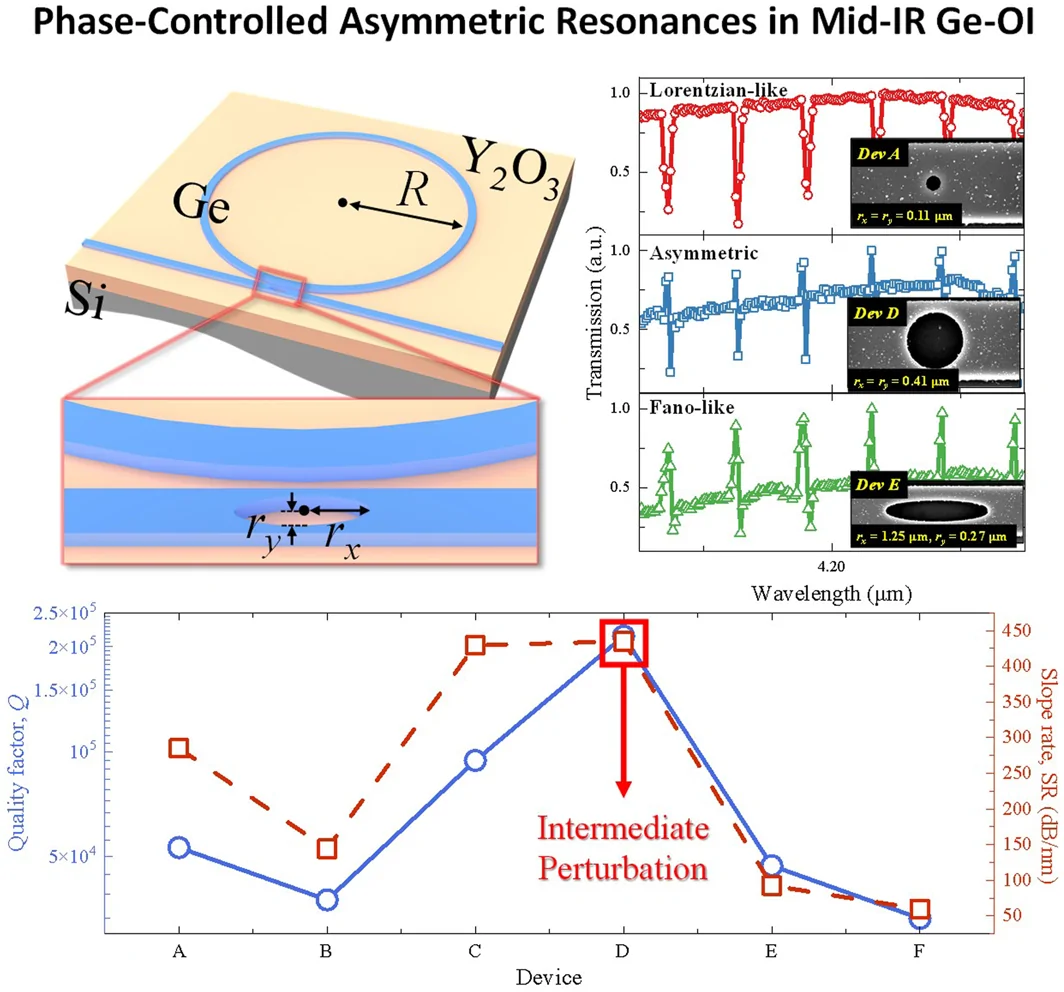

## 1 Introduction

The mid‐infrared (mid‐IR) spectral region has attracted significant interest in photonic integrated circuits (PICs) because it contains the fundamental vibrational absorption bands of a wide range of molecules, making it highly relevant to gas sensing [1], chemical analysis [2], environmental monitoring [3], and biomedical diagnostics [4]. In this spectral range, integrated photonic devices offer clear advantages over bulk free‐space systems in terms of footprint, stability, power consumption, and system‐level scalability. However, compact wavelength‐selective components that can effectively resolve and process spectral information on chips are needed to realize such systems. Because they offer strong spectral selectivity in a small footprint and are easily incorporated into larger photonic architectures, microring resonators (MRRs) play a key role in this matter. Owing to this versatility, MRRs have been widely explored for on‐chip interconnects [5, 6], signal processing [7, 8], nonlinear optics [9, 10], and spectroscopic sensing [11]. For spectroscopic sensing in particular, resonators with narrow linewidth and steep spectral response are highly desirable because both the quality factor *Q* and the transmission slope directly influence spectral discrimination and readout sensitivity. This importance is reflected in previous demonstrations of high‐*Q* mid‐IR MRRs on Si and III–V platforms [12, 13, 14], which have highlighted the promise of low‐loss resonant devices in this wavelength range while motivating continued exploration of alternative material systems.

Although these prior demonstrations highlight the strong potential of the existing mid‐IR platforms [15], the realization of high‐performance mid‐IR resonant devices still places stringent demands on the underlying material system in terms of transparency window, propagation loss, and optical confinement. In this context, germanium (Ge) has emerged as a promising platform because of its wide infrared transparency, high refractive index, and compatibility with CMOS‐compatible processing [16, 17]. These characteristics have also motivated extensive investigation of Ge‐based photonic platforms for mid‐IR applications, particularly for the realization of high‐*Q* MRRs and ring‐filter architectures that require both low loss and strong optical confinement [18, 19, 20, 21, 22]. In our previous work, we established a germanium‐on‐insulator (Ge‐OI) platform employing an yttria (Y_2_O_3_) buried insulator and demonstrated key passive and active device elements for mid‐IR PICs, including low‐loss waveguides [23], thermally tunable ring filters [24], gas‐sensing circuits [25, 26], and waveguide‐integrated photodetectors [27]. Collectively, these results support Ge‐OI as a scalable platform for implementing the essential passive and active building blocks required for mid‐IR PICs.

Beyond reducing propagation loss and increasing the *Q* of resonant photonic devices, engineering the resonance line shape itself provides an additional degree of freedom for improving device performance. Conventional MRRs exhibit symmetric Lorentzian resonances, whereas many filtering and sensing applications benefit from asymmetric resonances with a steeper local spectral transition. Fano resonance [28, 29, 30] is a representative example of such an asymmetric response and can be interpreted using a classical coupled‐oscillator model [31], in which a discrete resonant state interferes with a continuum‐like background pathway. In a side‐coupled waveguide–ring system, the asymmetric response can be generated by introducing an additional phase control in the bus waveguide transmission path. Previous studies [32, 33, 34, 35] have shown that an air hole inserted near the coupling region can induce a local phase shift and thereby transform the resonance from a Lorentzian to a Fano‐type line shape. Recent studies have demonstrated that engineered coupling, resonance hybridization, and symmetry breaking in resonant metasurfaces can provide high‐*Q* and strongly tunable spectral responses for infrared sensing and spectroscopy [36, 37, 38]. In contrast, the present work implements a localized perturbation directly in the bus–ring coupling region of a guided‐wave Ge‐OI microring resonator at 4.2 μm and experimentally examines the associated evolution and trade‐off among resonance asymmetry, loaded *Q*, and spectral slope. Although the underlying air‐hole phase‐perturbation mechanism is not unique to the mid‐IR, its implementation at 4.2 μm provides a useful design advantage. The larger optical mode size allows the air‐hole perturbation to be defined with larger absolute dimensions while remaining subwavelength with respect to the guided mode. This relaxes fabrication sensitivity and broadens the accessible phase‐shift range using a single localized perturbation, while maintaining compatibility with high‐*Q* Ge‐OI resonators.

In this work, we demonstrated phase‐engineered asymmetric resonances in a Ge‐OI MRR operating at 4.2 μm by introducing a subwavelength air hole into the bus‐waveguide coupling region to act as a localized phase perturbation. We analyzed the change in effective refractive index and the corresponding estimated phase shift as a function of hole geometry, and used an analytical phase‐modified transmission model [34, 39, 40, 41] to describe the resulting resonance evolution. To quantify the measured spectra of our Ge‐OI MRRs, the resonance line shapes were further analyzed using a weighted Fano–Lorentz line‐shape fitting framework [42, 43]. The resulting device series identifies an intermediate perturbation regime in which favorable combination of high Q and high spectral slope is observed, rather than at the largest air‐hole‐induced phase shift, with *Dev D* yielding a loaded quality factor of 2.15 × 10^5^ and a slope rate of 435 dB/nm. These results establish air‐hole‐assisted local perturbation as a practical design strategy for high‐*Q* and high‐slope mid‐IR resonant PIC components on the Ge‐OI platform. This approach could be promising for compact filtering and spectroscopic read‐out systems requiring sharp and tunable spectral responses.

## 2 Design and Fabrication

Fano‐type asymmetric resonances are conventionally described using the original Fano formalism, in which the line shape arises from interference between a discrete resonant state and a continuum‐like transmission pathway and is parameterized by a single asymmetry factor, *q* [28, 44]. Although this description is useful for idealized asymmetric resonances, a more general formulation is often required for first‐order resonant systems whose measured spectra are not fully captured by a single‐parameter Fano profile. As the phase shift introduced into the continuum transmission path increases, the measured resonances exhibit not only stronger asymmetry but also a systematic change in the residual Lorentzian character of the line shape. To describe this behavior, we analyze the measured asymmetric resonances using a weighted Fano–Lorentz model, in which the resonance is represented as a weighted superposition of Fano‐like and Lorentzian‐like components, allowing the asymmetry and the degree of Lorentzian admixture to vary independently [43]. This model does not alter the physical origin of Fano interference. Rather, it provides a more general and experimentally robust fitting framework for asymmetric spectra in first‐order resonant systems. In the present work, this weighted Fano–Lorentz model is used as a phenomenological framework to quantify the measured resonance line shapes. Accordingly, the fitted parameters *δ* and *η* are treated as empirical descriptors of asymmetry and Fano–Lorentz mixing, respectively, rather than as parameters with unique microscopic physical meanings. In this representation, the line‐shape evolution is more naturally interpreted as motion in the (*δ*, *η*) space, where the asymmetry factor *δ* denotes the degree and direction of the Fano‐like asymmetry, and the mixing factor *η* quantifies the relative weights of the Fano‐like and Lorentzian‐like components, rather than as variation of a single Fano parameter. This two‐parameter description follows the classification scheme proposed for first‐order resonant systems and provides a convenient way to track simultaneous changes in asymmetry and Lorentzian admixture. Figure 1a illustrates the proposed Ge‐OI MRR, in which an air hole is introduced into the bus‐waveguide coupling region to provide a localized phase perturbation in the transmission path. The device parameters are the ring radius *R*, the ring and bus waveguide widths *W*_ring_ and *W*_bus_, the coupling gap *G*, and the major and minor radii of the air hole, *r*_*x*_ and *r*_*y*_, respectively. The Ge thickness *H* was fixed at 500 nm, as determined by the epitaxially grown Ge layer used in the Ge‐OI platform. To estimate the air‐hole‐induced phase change, we first evaluated the effective refractive index in the perturbed coupling region, *n*_eff, C_, using an effective‐medium approximation as follows [35]:

(1) $$\begin{array}{c} n_{\text{eff},C}=n_{\text{eff},\text{WG}}+\frac{\pi r_{y}}{2W}\left(n_{\text{hole}}-n_{\text{eff},\text{WG}}\right) \end{array}$$

where *n*_hole_ is 1, *n*_eff,WG_ is the effective refractive index of the unperturbed Ge‐OI waveguide, and *W* is the waveguide width. For this Ge‐OI MRR, *W* was 1.2 μm, which is identical to both *W*_ring_ and *W*_bus_. At 4.2 μm, the calculated effective refractive index of the Ge‐OI waveguide, *n*_eff, WG_, was 2.76, as shown in the inset of Figure 1b. Based on this relation, *n*_eff, C_ was calculated as a function of hole geometry. Within this simplified effective‐medium approximation, the minor radius *r*_*y*_ primarily determines the effective‐index reduction in the air‐hole region, whereas the major radius *r*_*x*_ mainly contributes to the accumulated phase shift Δ*ϕ* through the perturbation length. Then, the corresponding Δ*ϕ* induced by the hole was defined by the following equation:

(2) $$\begin{array}{c} \Delta \phi =\frac{4\pi r_{x}}{\lambda }\left(n_{\text{eff},\text{WG}}-n_{\text{eff},C}\right) \end{array}$$

where Δ*ϕ* is the additional phase accumulated in the bus‐waveguide coupling section because of the effective‐index reduction introduced by the air hole. The calculated phase‐shift contour map is presented in Figure 1c as a function of *r*_*x*_ and *r*_*y*_. Because the waveguide width is 1.2 μm, *r*_*y*_ is geometrically constrained to remain below approximately 500 nm. In contrast, *r*_*x*_ can be increased over a wider range and therefore provides an effective means to increase the local phase shift. However, when the hole dimension becomes too large, the effective‐medium approximation becomes less reliable because the perturbation can no longer be treated as a weak local index perturbation. This approximation treats the air‐hole region as an effective perturbation averaged over the transverse waveguide width and is therefore most reliable when the hole primarily modifies the local propagation phase rather than acting as a strong scattering center. For larger elongated holes, the model provides a first‐order estimate of the phase shift, and quantitative deviations from the measured spectra may arise. Thus, the design range was selected to provide appreciable phase tunability while keeping the air‐hole perturbation within a regime where the effective‐index approximation remains physically meaningful.

**FIGURE 1:**
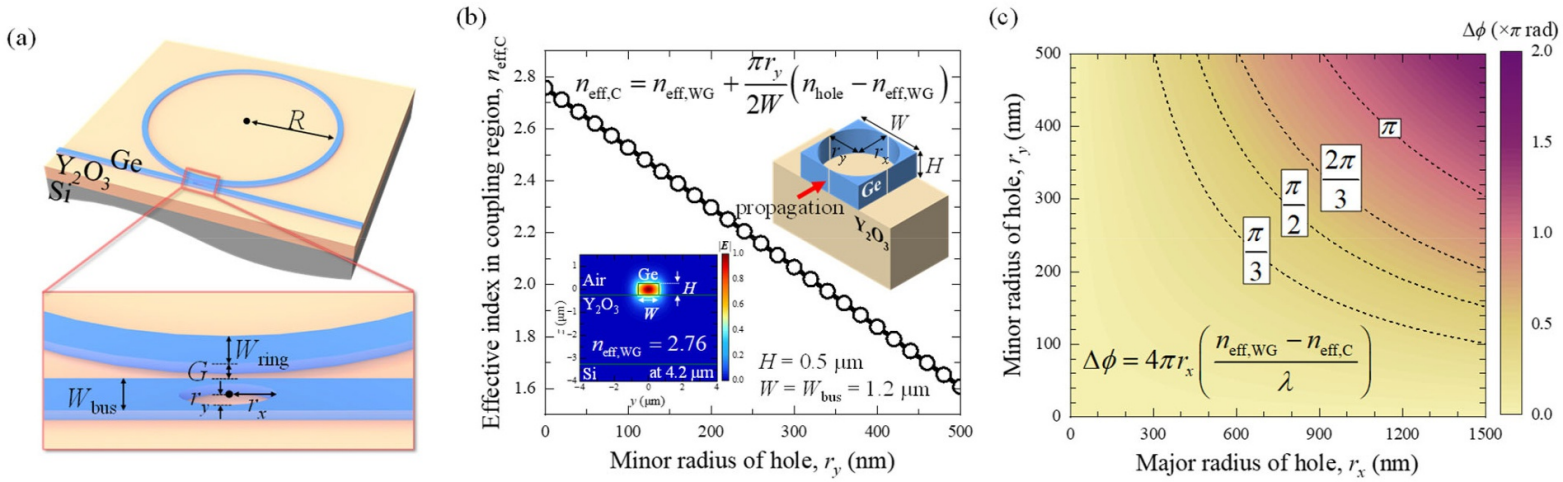
Device concept and phase‐perturbation mechanism. (a) Schematic of the Ge‐OI microring resonator (MRR) with an air hole introduced in the bus‐waveguide coupling region to serve as a local phase shifter for mid‐infrared operation. The radius of the MRR *R*, ring width *W*_ring_ and bus width *W*_bus_, coupling gap *G*, and major and minor hole radii *r*_*x*_ and *r*_*y*_ are indicated. (b) Calculated effective refractive index in the perturbed bus‐waveguide coupling region, *n*_eff,C_, as a function of *r*_*y*_, together with the calculated TE mode profile of a 1.2 μm‐wide Ge‐OI waveguide (*n*_eff,WG_ = 2.76) at *λ* = 4.2 μm. (c) Contour map of the calculated phase shift Δ*ϕ* induced by elliptical holes with different *r*_*x*_ and *r*_*y*_.

The air‐hole‐induced asymmetric resonance is described using two complementary analytical frameworks. The first is a phase‐modified transmission model *T* (*λ*) [34, 41] for the Ge‐OI MRR, where *T* (*λ*) denotes the normalized through‐port power transmission, and describes how a localized Δ*ϕ* introduced in the bus‐waveguide coupling section modifies the resonance spectrum. The second is a weighted Fano–Lorentz line‐shape model *F* (*λ*) [43], where *F* (*λ*) denotes the fitted transmission line shape used to characterize the resonance asymmetry and linewidth. In this way, the former provides a device‐level physical description of the phase‐controlled spectral evolution, whereas the latter provides an effective representation of the measured line shapes.

The transmission of the phase‐perturbed MRR is written as follows:

(3) $$\begin{array}{c} T\left(\lambda \right)={\left|te^{-i\Delta \phi }-\frac{\kappa_{1}\kappa_{2}{ae}^{\frac{i2\pi Ln_{\text{eff},\text{WG}}}{\lambda }}}{1-t{ae}^{\frac{i2\pi Ln_{\text{eff},\text{WG}}}{\lambda }}}\right|}^{2} \end{array}$$

where *t* is the self‐coupling coefficient, *κ*_1_ and *κ*_2_ are the cross‐coupling coefficients between the bus waveguide and the ring resonator, *a* is the round‐trip amplitude attenuation factor, and *L* is the round‐trip length of the resonator. Supporting Information S1: Figure S1 shows calculated spectra for selected Δ*ϕ* = π/3, π/2, 2π/3, π, 4π/3, and 5π/3. In this calculation, *t* = *a* = 0.95 was used with symmetric coupling, and *κ*_1_ = *κ*_2_ = (1*−t* ^2^)^1/2^. Equation (3) represents the phase‐dependent modification of the interference between the continuum transmission through the bus waveguide and the discrete resonance of the microring. The resulting spectra exhibit varying degrees of asymmetry, as well as a residual Lorentzian character, which motivates the use of a separate line‐shape model for quantitative characterization. In this reduced model, the coupling and round‐trip attenuation parameters are kept fixed to isolate the role of the local phase shift. In the fabricated devices, however, the air‐hole perturbation can also modify the bus–ring coupling coefficient and introduce additional scattering loss. Therefore, Δ*ϕ* values should be regarded as model‐based phase‐shift parameters estimated from the effective‐index approximation or spectral fitting, rather than as an independently measured quantity. This distinction is important when interpreting the measured linewidth, quality factor, and slope‐rate variations across the device series.

Although *T* (*λ*) explains how the air‐hole‐induced phase shift reshapes the resonance spectrum, quantitative comparison of the resulting asymmetric line shapes requires a separate fitting framework. For this, we employed the weighted Fano–Lorentz model,

(4) $$\begin{array}{c} F\left(\lambda \right)=A\left(\lambda \right)\left[\eta \frac{{\left(\varepsilon +\delta \right)}^{2}}{1+\varepsilon^{2}}+\left(1-\eta \right)\frac{1}{1+\varepsilon^{2}}\right] \end{array}$$

with the normalized detuning *ε* defined as follows:

(5) $$\begin{array}{c} \varepsilon =\frac{\lambda -\lambda_{0}}{\gamma } \end{array}$$

where *λ*_0_ is the resonance wavelength, *γ* is the half‐linewidth parameter, *δ* is the asymmetry factor, and *η* is the mixing factor. Here, *A* (*λ*) denotes a slowly varying background function introduced to account for the local transmission baseline within the fitting window. Following the same reasoning as in [43], where a linear dependence was used in the frequency domain, we expressed *A* (*λ*) in the wavelength domain and approximated it by a low‐order Taylor expansion. In the present fitting, terms up to second order were retained to capture the residual baseline curvature without modifying the underlying resonance model. From the fitted linewidth, the loaded quality factor is obtained as *Q* = *λ*_0_/(2*γ*). This fitting form is introduced not to replace the physical transmission model in Equation (3), but to provide a compact and generalized description of the measured resonance evolution in terms of the weighted Fano–Lorentz parameter space.

To provide a physically transparent interpretation of this generalized fitting space, we summarized the weighted Fano–Lorentz line‐shape landscape and its relation to the phase perturbation introduced by the air hole. Figure 2a presents the normalized steepness S* as a function of *δ* and *η*, calculated from *F* (*λ*). In this parameter space, S* serves as a compact metric for quantifying how sharply the transmission changes near resonance. Here, S* denotes the maximum absolute slope of the normalized line shape with respect to the normalized detuning, defined as S* = max_*ε*_|*dF*/*dε*|, and is used as a compact metric for evaluating the steepest locally accessible spectral transition. The resulting contour map shows that the achievable steepness is governed by the combined evolution of *δ* and *η*, thereby motivating the use of *F* (*λ*). Representative line shapes for selected (*δ*, *η*) combinations are shown in the inset, illustrating the gradual transition from Lorentzian‐dominant to strongly asymmetric responses.

**FIGURE 2:**
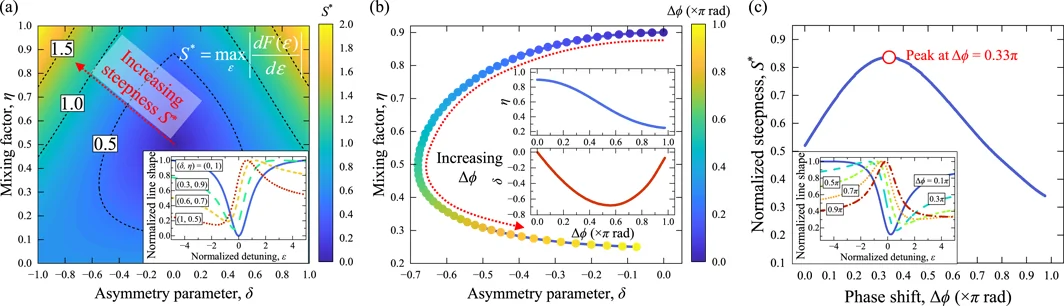
Conceptual interpretation of the weighted Fano–Lorentz line‐shape space. (a) Contour map of the normalized steepness S* as a function of asymmetry parameter *δ* and mixing factor *η*, calculated from the weighted Fano–Lorentz model *F* (*ε*). The symmetry relation *F* (*ε*, *η*, –*δ*) = *F* (–*ε*, *η*, *δ*) leads to a *δ*‐symmetric steepness landscape. Inset: representative normalized line shapes for selected (*δ*, *η*) combinations. (b) Idealized phase trajectory in the (*δ*, *η*) space obtained by generating phase‐modified transmission spectra *T* (*λ*, Δ*ϕ*) from the analytic Ge‐OI air‐hole‐induced MRR model at *λ* = 4.2 μm, using the calculated effective indices, fixed ring radius, and *t* = *a* = 0.95, subsequently refitting them with the weighted Fano–Lorentz expression. Inset: the corresponding one‐dimensional evolutions of *δ* and *η* along the phase trajectory. (c) Calculated normalized steepness S* parameterized by Δ*ϕ* for the Ge‐OI air‐hole‐induced MRR at 4.2 μm, obtained by mapping the (*δ*, *η*) trajectory in (b) onto the weighted Fano–Lorentz steepness landscape, showing a maximum at Δ*ϕ* = 0.33*π*. Inset: representative normalized line shapes for selected phase‐shift values.

Having established the weighted Fano–Lorentz steepness landscape in Figure 2a,b links this abstract parameter space to the device‐level phase perturbation. Using the analytical phase‐modified transmission model *T*(*λ*) with the Ge‐OI MRR parameters fixed at their design values, idealized spectra were generated as a function of Δ*ϕ* at 4.2 μm under fixed coupling conditions and were subsequently fitted using the weighted Fano–Lorentz form *F*(*λ*). The resulting phase trajectory in the (*δ*, *η*) space shows how phase‐controlled interference drives the resonance through different line‐shape regimes. Importantly, this trajectory is introduced as a model‐based interpretive tool that bridges the geometrically induced phase perturbation and the experimentally extracted fitting parameters. In this sense, the resonance evolution is mapped onto the (*δ*, *η*) space, where the simultaneous evolution of asymmetry and Lorentzian admixture can be tracked explicitly. The inset further shows the corresponding one‐dimensional evolution of *δ* and *η* as the phase shift increases. Figure 2c shows S* depending on Δ*ϕ*, obtained by projecting the phase trajectory in Figure 2b onto the weighted Fano–Lorentz steepness landscape in Figure 2a. A clear maximum is found near Δ*ϕ* = 0.33π, indicating that, in the present Ge‐OI MRR, the model‐based physical trajectory provides a useful design parameter for linking air‐hole geometry to local spectral steepness. The inset shows representative normalized line shapes at selected phase‐shift values, further illustrating how phase tuning modifies the spectral transition. In the present work, the significance of our analysis is therefore not simply that the steepest response occurs at an intermediate phase condition, but that the local phase shift induced by the air hole serves as the key design variable linking air‐hole geometry to resonance steepness in the Ge‐OI mid‐IR MRR. The effective‐index perturbation mechanism itself is not unique to the mid‐IR, but the accessible design space for single‐hole phase engineering becomes more favorable at longer wavelengths. Although Equation (2) implies that phase accumulation becomes less efficient as the wavelength increases, the longer wavelength simultaneously allows larger absolute air‐hole geometries to remain deeply subwavelength and less susceptible to strong scattering. Consequently, the air hole can function more reliably as a local effective‐index perturbation rather than as a discrete scattering defect, which widens the usable geometry range for effective‐medium‐theory‐like phase tuning and enables a large accessible phase shift with only a single hole in the present Ge‐OI MRR. Therefore, this analysis provides a design basis for selecting the air‐hole geometry and the corresponding phase‐shift regime for achieving steep spectral response on the Ge‐OI platform.

Based on the phase‐engineered device design, Ge‐OI MRRs were fabricated on our Ge‐OI platform optimized for low‐loss mid‐IR operation. The detailed wafer fabrication procedure for the Ge‐OI MRR has been reported previously [19], and only the key steps relevant to the present devices are summarized here. A chemically cleaned Si (100) substrate and a Ge epitaxial wafer grown on Si, consisting of a 900 nm Ge strained‐relaxed buffer, a 10 nm Si_0.5_Ge_0.5_ layer, and a 500 nm Ge device layer, were prepared. After removal of the native oxide, a 1.5 μm‐thick Y_2_O_3_ layer was deposited on both wafers by RF sputtering. The Y_2_O_3_ bonding surfaces were subsequently planarized by chemical mechanical polishing using a silica colloidal slurry to achieve subnanometer surface roughness suitable for direct wafer bonding. After surface cleaning and O_2_ plasma activation, the Si and Ge epitaxial wafers were directly bonded under heating and mechanical pressure in vacuum. The unwanted epitaxial layers on the epitaxial side were then selectively removed through sequential mechanical grinding of the Si substrate, wet etching of the remaining Si and Si_0.5_Ge_0.5_ using diluted tetramethylammonium hydroxide, and removal of the Ge strained relaxed buffer using an ammonia–peroxide mixture. This process yielded the Y_2_O_3_‐insulated Ge‐OI wafer used for the mid‐IR photonic devices. The MRR patterns with the holes were then defined by electron‐beam lithography, followed by ICP‐RIE in a C_4_F_8_/SF_6_ plasma. Finally, the residual resist was removed by acetone cleaning and plasma treatment. Figure 3a summarizes the overall fabrication flow.

**FIGURE 3:**
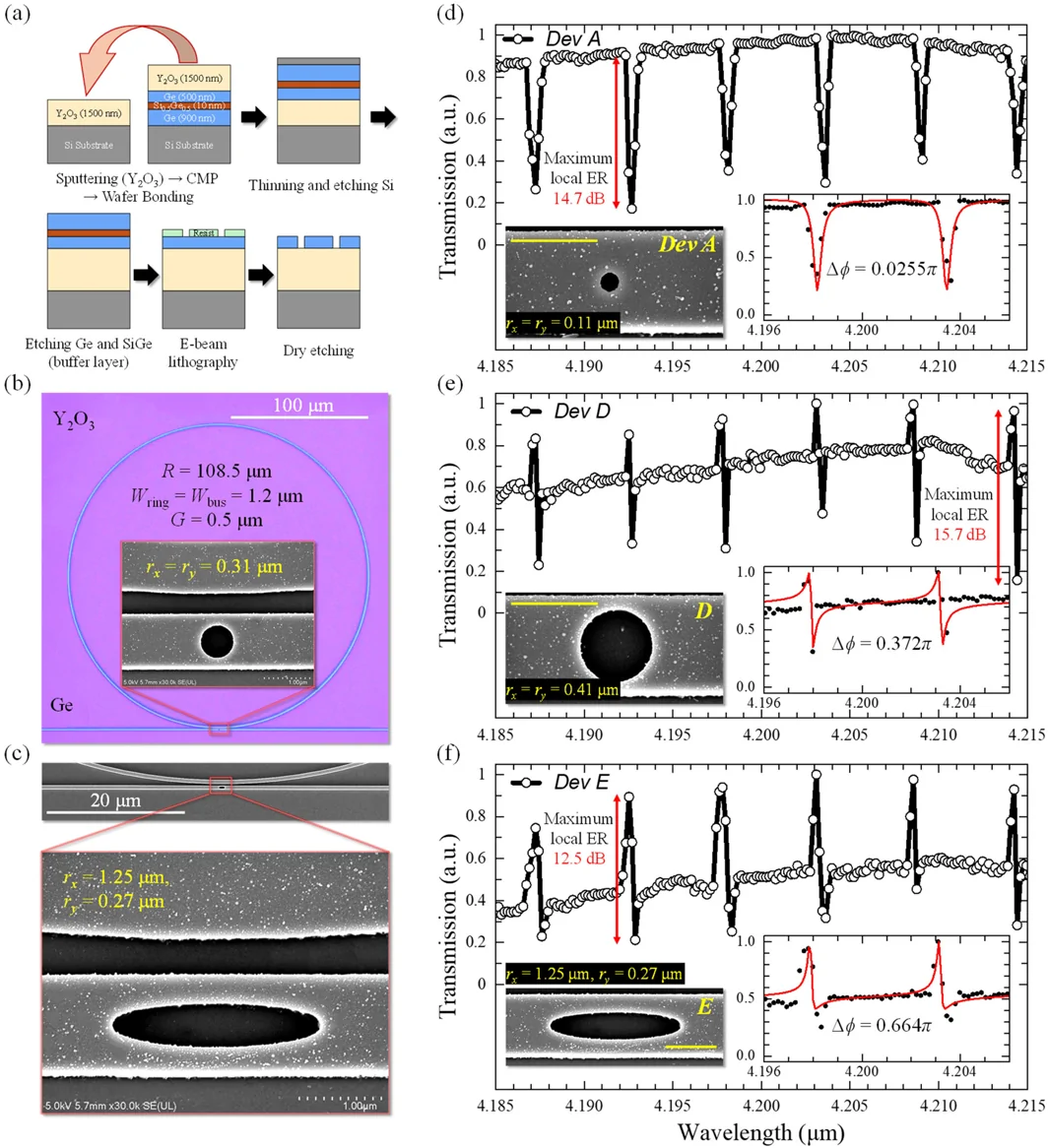
Fabrication and measured spectral evolution of the Ge‐OI air‐hole‐assisted MRR devices. (a) Fabrication process flow of the Ge‐OI MRR with an air‐hole phase shifter. (b) Optical microscope image of a fabricated asymmetric‐resonance Ge‐OI MRR, showing the ring resonator and coupling region; inset: SEM image of a representative circular‐hole device. (c) SEM images of representative devices with enlarged perturbation geometries, illustrating the systematic increase in the elliptical‐hole size. (d–f) Measured normalized transmission spectra of representative devices (*Dev A*, *D*, and *E*), including their SEM images (lower left) of the corresponding coupling regions and analytical fitting curves obtained from the phase‐modified transmission model by *T* (*λ*, Δ*ϕ*).

An optical microscope image of a fabricated Ge‐OI MRR is shown in Figure 3b. The device consists of a ring resonator with *R* = 108.5 μm, *W*_ring_ = *W*_bus_ = 1.2 μm, and *G* = 0.5 μm, together with a locally patterned air hole in the coupling region. All six measured devices share the same MRR geometry, whereas the air‐hole radii were systematically varied. *Dev A–D* correspond to circular air‐hole perturbations with *r*_*x*_ = *r*_*y*_ = 0.11, 0.21, 0.31, and 0.41 μm, respectively. *Dev E* and *Dev F* employ elongated elliptical air‐hole perturbations with (*r*_*x*_, *r*_*y*_) = (1.25, 0.27) μm and (1.50, 0.30) μm, respectively. These geometries are summarized in Supporting Information S1: Table S1. The inset SEM image confirms the formation of a representative circular‐hole perturbation in the bus waveguide. The designed air‐hole dimensions are well within the practical fabrication range of our optimized electron‐beam lithography and dry‐etching process. Figure 3c further shows SEM images of representative devices with larger elliptical‐hole geometries, demonstrating that both circular and elongated perturbation patterns were reliably realized on the Ge‐OI platform. These images verify that the hole geometry can be systematically controlled over the design range required for phase engineering of the resonance line shape.

## 3 Results and Discussion

The measured spectra of the fabricated devices are presented in Figure 3d–f for three representative resonators, *Dev A*, *D*, and *E*, which correspond to weak, intermediate, and strong perturbation regimes, respectively. Additional devices with various hole sizes were also fabricated and measured, as summarized in Supporting Information S1: Figure S2. The spectra were obtained using our mid‐IR waveguide characterization setup [19], where N_2_ purging was employed to suppress atmospheric water and CO_2_ absorption at 4.2 μm. The broad‐range spectra in Figure 3 were acquired with a wavenumber step of 0.1 cm^−1^ and were independently normalized to the maximum transmission within each measured window. In contrast, the high‐resolution spectra used for the fitting analysis in Figure 4 and Fig. S3 were acquired with a finer wavenumber step of 0.025 cm^−1^, corresponding to approximately 0.05 nm near 4.2 μm, using a timing‐triggered acquisition scheme with optimized time constant and recording time. These high‐resolution spectra are presented using the measured detector output in mV. The EC‐QCL linewidth was below 100 MHz. Because the apparent extinction ratio (ER) depends on the sampling density and on whether the local maximum and minimum around the resonance are fully resolved, the ER values indicated in Figure 3 and those extracted from the high‐resolution fitting analysis are reported separately. The resonance line shape gradually changed from a nearly symmetric Lorentzian‐like behavior to a significantly asymmetric Fano‐like response as the small hole expanded from a tiny circular structure to an extended elliptical hole. This trend was consistent with the increase in the hole‐induced phase shift predicted from the effective‐index analysis in Figure 1c and with the phase trajectory in the weighted Fano–Lorentz parameter space shown in Figure 2b. The red arrows indicate the maximum local ER extracted around the selected resonance of each representative device, providing an additional measure of local transmission contrast. These results support the interpretation that the local air hole effectively controls the interference condition in the Ge‐OI MRR.

**FIGURE 4:**
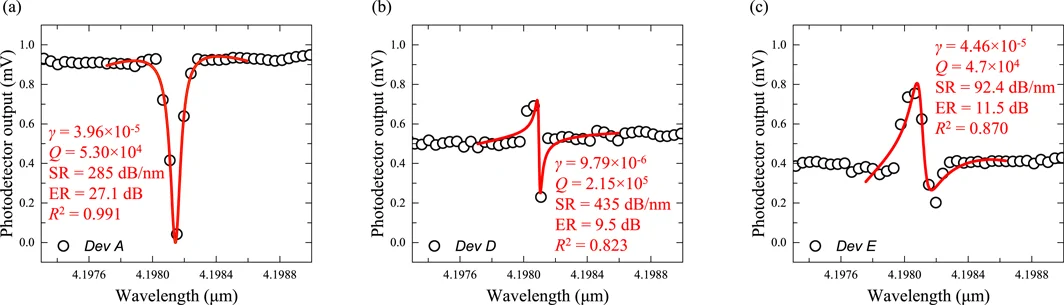
Weighted Fano–Lorentz fitting of representative measured resonances. (a–c) High‐resolution measured spectra (black circles) and fitted weighted Fano–Lorentz curves (red lines) for *Dev A*, *D*, and *E* spanning different line‐shape regimes. The extracted linewidth parameter *γ,* loaded quality factor *Q* (= *λ*_0_/(2*γ*)), slope rate (SR), extinction ratio (ER), and coefficient of determination *R* ^2^ are indicated in each graph. The spectra are presented as the unnormalized photodetector output in mV and were directly used for the weighted Fano–Lorentz fitting. For SR extraction, only the fitted line shape was subsequently normalized to its local maximum; the corresponding normalized representation of the same high‐resolution datasets is shown in Supporting Information S1: Figure S3.

For *Dev A* (*r*_*x*_ = *r*_*y*_ = 0.11 μm), the estimated phase shift Δ*ϕ* was only 0.026π, which was close to zero, and the resonance remained near the symmetric limit. In this regime, the phase shift was too weak to drive the resonance far from a Lorentzian response, although a small asymmetry was still observable. Based on our previous characterization of unperturbed MRRs with identical waveguide geometry [19], this weakly perturbed state is consistent with operation near the critical‐coupling condition. As the air‐hole dimensions increase, the perturbation can reduce the effective bus‐to‐ring coupling strength and introduce additional scattering loss. Therefore, the simultaneous increase in loaded *Q* and decrease in the ER from *Dev A* to *Dev D* suggest a gradual shift toward a more undercoupled condition, which can contribute to the observed increase in the loaded *Q* factor in addition to the phase‐induced line‐shape asymmetry. Accordingly, the experimentally observed variations in loaded *Q* and SR should be interpreted as the combined consequence of phase perturbation, modification of the bus–ring coupling condition, and additional scattering loss, rather than as an effect of phase shift alone. As the hole geometry increased to *Dev D* (*r*_*x*_ = *r*_*y*_ = 0.41 μm), the Δ*ϕ* reached 0.37π, and the spectral response became markedly asymmetric, accompanied by a substantially steeper local transition. Particularly, the phase shift of this device lies close to the optimum region predicted in Figure 2c, where the normalized steepness reaches a maximum near 0.33π. Thus, *Dev D* supports an intermediate phase shift rather than the greatest one available, which gives the best conditions for enhancing spectral steepness.

A further increase in phase shift was observed in *Dev E*, which incorporated an elliptical hole (*r*_*x*_ = 1.25 μm, *r*_*y*_ = 0.27 μm) and yielded Δ*ϕ* = 0.66π. In this case, the line shape became more strongly distorted and clearly Fano‐like, confirming that the elongated hole geometry extends the accessible phase‐shift range without increasing *r*_*y*_ beyond the geometric limit imposed by the 1.2 μm‐wide waveguide. However, the spectra also show that a larger phase shift does not necessarily lead to better resonance discrimination or greater spectral steepness. Instead, once the resonance moves beyond the optimal region of the phase–steepness map, the line shape becomes more strongly skewed but less favorable for a steep local spectral transition. The analytical fitting curves reproduce the measured spectra well across all three representative devices, supporting the interpretation that the observed line‐shape evolution is strongly correlated with the geometry‐controlled phase shift in the bus‐waveguide path. This nonmonotonic behavior is also reflected in the local extinction ratio around the selected resonance: the maximum local ER increases from 14.7 dB in *Dev A* to 15.7 dB in *Dev D*, but then decreases to *12.5 dB* in *Dev E*, indicating that a more strongly distorted Fano‐like resonance does not necessarily provide larger local transmission contrast. This trend further supports the conclusion that the optimum operating regime is reached at an intermediate phase perturbation, where linewidth, asymmetry, and resonance contrast are most favorably balanced. Therefore, these results show that hole‐assisted phase control enables systematic and predictable tuning of the resonance line shape in Ge‐OI MRRs, with the most favorable regime for steep spectral discrimination occurring at an intermediate phase shift rather than at the maximum accessible value.

Subsequently, the transmission spectra around the selected central resonance were fitted using Equation (4) to quantify the experimentally observed line‐shape evolution (Supporting Information S1: Figure S3). The slope rate (SR) was evaluated from the fitted line shape rather than from point‐to‐point differentiation of the raw measurement data. Specifically, the fitted transmission curve was first normalized to its local maximum, and the maximum absolute derivative |*dT*_fit, norm_/*dλ*| was identified within the selected resonance window. The corresponding slope was then converted to dB/nm as SR = (10/ln10) |*dT*_fit, norm_/*dλ*|/*T*_fit, norm_ at the maximum‐slope wavelength. Because the extracted *Q*, SR, and weighted Fano–Lorentz parameters were obtained from nonlinear line‐shape fitting, the coefficient of determination *R* ^2^ is reported in Supporting Information S1: Table S1 for each device to indicate the fitting agreement. The reported *R* ^2^ values provide a reference for evaluating the reliability of the extracted fitting‐based parameters. Because the high‐*Q* resonances are sampled with a finite wavelength interval, the fitted linewidth, *Q*, and SR should be regarded as fitting‐derived estimates, particularly for spectra with moderate fitting agreement. Accordingly, small quantitative differences between devices are not interpreted as statistically significant, and the extracted values are primarily used to describe the overall line‐shape and performance trends across the device series. For *Dev D*, the fitted SR of 435.1 dB/nm corresponds to a relatively broad 95% confidence interval of 305.2–684.9 dB/nm, reflecting the fitting sensitivity of its narrow resonance under finite spectral sampling. The reported values are therefore interpreted conservatively as fitted point estimates. The residual‐bootstrap analysis quantifies fitting and spectral‐sampling uncertainty under the selected line‐shape model and does not account for device‐to‐device fabrication variations. Nevertheless, the residual‐bootstrap distributions remain centered around the reported fitted *Q* and SR values, supporting their use as reasonable fitting‐derived estimates under the present spectral‐sampling conditions (Supporting Information S1: Table S2 and Figures S4 and S5). Figure 4a–c shows representative fitting results for *Dev A*, *D*, and *E*, respectively. In all three cases, the fitting reproduces the measured spectral features with reasonable agreement, supporting the use of *F*(*λ*). For *Dev A*, the fitted linewidth parameter *γ* was 3.96 × 10^−5^, corresponding to a *Q* of 5.30 × 10^4^, with an SR of 285 dB/nm, where *R* ^2^ was 0.991. This device remained close to the Lorentzian regime because the phase shift was relatively weak, and its spectral transition, although asymmetric, was still comparatively broad. In contrast, *Dev D* exhibited a substantially reduced fitted *γ* of 9.79 × 10^−6^, corresponding to the highest fitted *Q* of 2.15 × 10^5^ among the representative devices, together with a high SR of 435 dB/nm. According to these results, *Dev D* provided the most favorable operating point for achieving strong spectral discrimination among the devices examined. This behavior is consistent with the phase‐to‐steepness analysis introduced in Figure 2c. The estimated phase shift for *Dev D* lies near the predicted optimum region, where the weighted balance between asymmetry and Lorentzian admixture maximizes the local spectral steepness. In other words, the best performance is achieved not by the strongest possible phase shift, but by an intermediate phase condition that sharpens the spectral transition while preserving a sufficiently narrow linewidth.

Although *Dev E* showed a more strongly distorted Fano‐like response, its fitted linewidth increased to *γ* = 4.46 × 10^−5^, and the corresponding *Q* and SR decreased to 4.70 × 10^4^ and 92.4 dB/nm, respectively. This means that increasing the phase shift beyond the optimum range can drive the resonance away from the most favorable region of the weighted Fano–Lorentz parameter space, yielding a highly asymmetric yet less steep spectral response. More generally, devices with larger air holes and stronger phase shifts exhibited increasingly distorted resonance profiles. It should be noted that the data sampling density in these steeply varying edge regions is constrained by the inherent step resolution of currently available mid‐IR tunable lasers. Despite this practical hardware limitation, the weighted Fano–Lorentz model still demonstrates generally good agreement with the measured spectra. With moderate‐to‐high *R* ^2^ values across the full devices (Supporting Information S1: Table S1), the extracted parameters retain clear physical relevance and consistently capture the systematic line‐shape evolution. Notably, the strongest perturbation does not maximize either SR or the local ER, indicating that excessive phase distortion enhances asymmetry at the expense of useful spectral sharpness and transmission contrast. As a result, localized air‐hole phase engineering provides a viable route to tuning Ge‐OI MRRs toward an optimal high‐*Q*, high‐slope operating regime, and the weighted Fano–Lorentz model offers an effective framework for comparing the resulting line‐shape evolution and linewidth characteristics.

In Figure 5a, the phase shifts estimated from spectral fitting follow the same overall trend as the theoretical values calculated from the effective‐index‐based phase model. Although quantitative deviations remain for *Dev E* and *F*, the overall increase in phase shift with increasing hole perturbation is captured by the spectral‐fit estimates, supporting the interpretation that the local air hole acts as an effective phase‐control element in the bus‐waveguide coupling region. The extracted weighted Fano–Lorentz parameters are summarized in Figure 5b. As the hole geometry increases, the measured resonances evolve systematically through the (*δ*, *η*) parameter space. From *Dev A* to *Dev E*, *δ* shifts progressively toward more negative values, whereas *η* decreases overall, indicating that the resonance moves away from the nearly Lorentzian regime and into increasingly asymmetric line‐shape states within the weighted Fano–Lorentz space. This behavior is consistent with the phase‐trajectory picture introduced in Figure 2b and further supports the interpretation that the measured line‐shape evolution is effectively captured in the (*δ*, *η*) space. *Dev F* deviates from the monotonic trend and appears in a different quadrant of the parameter space, suggesting that once the phase shift becomes sufficiently large, the resonance can enter a distinct interference regime rather than simply continuing the earlier progression.

**FIGURE 5:**
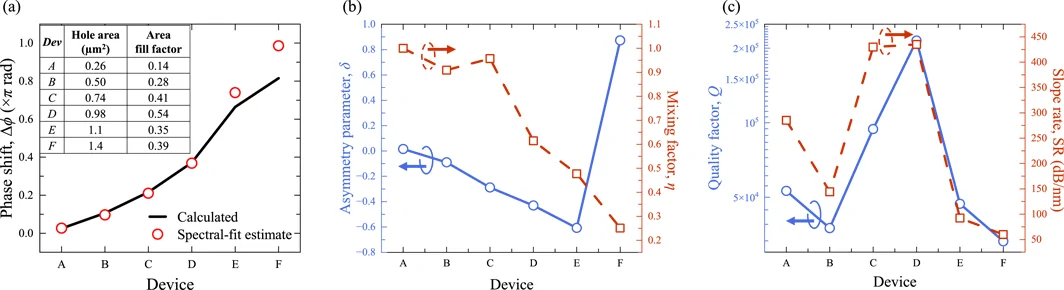
Extracted weighted Fano–Lorentz parameters and performance metrics across the device series. (a) Comparison between effective‐index‐calculated Δ*ϕ* and spectral‐fit‐estimated Δ*ϕ* for *Devices A–F*. (b) Evolution of *δ* and *η*, showing the systematic movement of the measured resonances through the weighted Fano–Lorentz parameter space. (c) Loaded quality factor *Q* and SR extracted from the fitted spectra.

The corresponding performance metrics are shown in Figure 5c. The fitted *Q* and SR values are both high in the intermediate phase‐shift regime, with *Dev D* exhibiting the most favorable fitted combination of the two metrics among the examined devices. This result is consistent with the phase‐to‐steepness analysis in Figure 2c and with the representative fitting results in Figure 4. Among the examined devices, the most favorable spectral discrimination is observed in an intermediate perturbation regime, where linewidth narrowing and asymmetric steepening are simultaneously optimized. For larger perturbations, such as those represented by *Dev E* and *F*, the resonance remains strongly asymmetric but no longer provides the highest *Q* or SR, indicating that excessive phase shift can move the device away from the optimum high‐slope operating condition. Consequently, these results establish that the air hole acts as a systematically tunable phase‐control element that governs not only the onset of asymmetric resonance formation but also the accessible high‐*Q*, high‐slope operating regime of the Ge‐OI mid‐IR MRR. To further place the present device performance in context, a benchmarking summary against previously reported resonant photonic devices is provided in Supporting Information S1: Table S3.

## 4 Conclusion

In conclusion, we have demonstrated a phase‐engineered asymmetric MRR based on a Ge‐OI platform at 4.2 μm by introducing a subwavelength air hole as a localized phase shifter in the bus‐waveguide coupling region. This simple geometry enables deterministic control of the resonance line shape, driving the device from a nearly Lorentzian response into a weighted Fano–Lorentz regime through geometry‐dependent phase tuning. Effective‐index‐based phase analysis, analytical transmission modeling, and experimental fitting collectively show that the resulting resonance evolution is governed by a tunable trajectory in the (*δ*, *η*) parameter space. The extracted device series further identifies an intermediate perturbation regime that provides the most favorable combination of a fitted *Q* of 2.15 × 10^5^ and an SR of 435 dB/nm among the measured devices. Beyond demonstrating asymmetric resonance control in a mid‐IR Ge‐OI MRR, this work establishes air‐hole‐induced phase perturbation as a practical design strategy for engineering high‐*Q*, high‐slope spectral responses in integrated mid‐IR photonic devices. In particular, the broad mid‐IR transparency and fabrication compatibility of the Ge‐OI platform make it well suited for implementing subwavelength‐scale local phase shifters and compact wavelength‐selective resonant components in this spectral window. Importantly, operation at 4.2 μm provides a practical design window in which a single air‐hole perturbation can provide appreciable phase tunability while remaining subwavelength with respect to the guided mode and compatible with high‐*Q* Ge‐OI resonator operation. Hence, this result highlights an effective strategy for compact wavelength‐selective components for mid‐IR PICs, where sharp and controllable resonance features are important for spectral filtering, signal processing, spectroscopic sensing, and, more broadly, integrated photonic functionalities including on‐chip interconnects and nonlinear optics.

## Author Contributions

**Jinha Lim:** conceptualization, methodology, investigation, validation, writing – original draft, writing – review and editing. **SangHyeon Kim:** conceptualization, supervision, resources, writing – review and editing, funding acquisition.

## Supporting information


Supporting Information S1

## Data Availability

The data that support the findings of this study are available from the corresponding author upon reasonable request.
