# Supplementary material for: Phase‐Controlled Asymmetric Resonances for Enhanced Spectral Slope in a Ge‐OI Microring Resonator at 4.2 μm
Source: Nanophotonics. 2026 Oct 14;15(20):e70321. doi: 10.1002/nap2.70321 (PMC13669529; doi:10.1002/nap2.70321)
Supplement: Supplementary file 1 [file NAP2-15-e70321-s001.docx]

**Supplementary Information**

**Title: Phase-Controlled Asymmetric Resonances for Enhanced Spectral Slope in a Ge-OI Microring Resonator at 4.2 μm**

Figure S1. Simulated normalized transmission spectra of the microring resonator for phase shifts Δ*ϕ* ranging from (1/3)*π* to (5/3)*π*, showing the evolution from a Lorentzian to an asymmetric Fano lineshape.

Figure S1 shows simulated transmission spectra to illustrate the localized phase perturbation for selected phase shifts ranging from Δ*ϕ* = π/3 to 5π/3. The spectra show that the resonance evolves systematically with increasing phase shift for asymmetric Fano-like characteristics. Especially, the spectral evolution is not limited to a simple increase in asymmetry magnitude but also includes reversal of the line-shape orientation and variation in the residual Lorentzian component depending on the phase condition. This trend supports the interpretation that the air hole acts as a local phase-control element in the bus-waveguide path and provides the intuitive spectral basis for the phase trajectory and steepness analysis.

Figure S2. Measured normalized transmission spectra of Ge-OI microring resonators with different air-hole geometries in the bus-waveguide coupling region over 4.185–4.215 μm. Left insets: SEM images of the corresponding hole structures for *Dev A*–*F*. Right insets: enlarged views of the selected resonances with analytical fitting curves from the phase-modified transmission model.

To examine the overall line-shape evolution across the full device series, the measured transmission spectra of *Dev A*–*F* are summarized in Fig. S2. As the air-hole geometry increases from a small circular perturbation to a larger circular and eventually elongated elliptical shape, the resonance progressively departs from a nearly Lorentzian response and develops increasingly asymmetric Fano-like line shape. The corresponding SEM images confirm the systematic geometric variation in the coupling region, while the inset fits show that the analytical phase-modified transmission model consistently reproduces the selected resonances across the series.

Figure S3. High-resolution transmission spectra near the selected resonance for *Dev A*–*F* with weighted Fano–Lorentz fitting curves. The extracted quality factor *Q*, extinction ratio (ER), and slope rate (SR) at a slope point are indicated for each device.

Figure S3 shows magnified views of the selected resonances for *Dev A*–*F* with the weighted Fano–Lorentz fitting curves used for quantitative analysis. The fitting captures the measured line shapes reasonably well across the full device series, despite the increasing distortion observed in the more strongly perturbed devices. From these fits, the loaded quality factor, extinction ratio (ER), and maximum slope point were extracted and compared among the devices. The results show that the spectral response evolves non-monotonically with perturbation strength. Although stronger phase perturbation generally increases the degree of asymmetry, it does not necessarily improve the useful spectral metrics simultaneously. In particular, the maximum slope is achieved for the intermediate devices, whereas the ER and line-shape distortion follow different trends, supporting the conclusion that the optimum operating regime occurs at an intermediate phase perturbation rather than at the largest hole geometry.

Table S1 Extracted weighted Fano–Lorentzian fitting parameters and resonance metrics for six Ge-OI MRR devices

Table S1 summarizes the weighted Fano–Lorentzian fitting parameters extracted from the measured resonance spectra of six Ge-OI MRRs with different air-hole geometries. In addition to the geometric parameters (*r_x_*, *r_y_*), the fitted line-shape parameters *δ* and *η*, the mapped phase shift *ϕ*, and the goodness-of-fit *R*^2^ are listed together with the extracted resonance metrics, including the resonance wavelength *λ*_0_, linewidth parameter *γ*, ER, *Q* factor, and slope rate (SR). The unbracketed ER values are extracted from the high-resolution weighted Fano–Lorentz fits in Fig. S3, whereas the values in parentheses indicate larger local ER values observed at other resonance dips in the broader spectra of Fig. S2. The table shows that the evolution of the resonance response across the device set is consistently captured by the weighted Fano–Lorentzian framework, while also revealing substantial device-to-device variation in asymmetry, *Q* factor, and spectral slope. In particular, the comparison highlights that the largest SR values are not determined by *Q* alone but arise from the interplay between resonance linewidth and line-shape asymmetry.

Figure S4. Residual-bootstrap distributions of the fitted loaded quality factor *Q* for *Dev A*–*F*. Each histogram shows the distribution obtained from 5,000 conditional residual-bootstrap realizations. The solid vertical line indicates the reported fitted *Q*, while the dashed vertical lines indicate the 2.5th and 97.5th percentiles defining the 95% fitting-derived confidence interval (CI).

Figure S5. Residual-bootstrap distributions of the fitted SR for *Dev A*–*F*. SR was recalculated from the fitted line shape for each of the 5,000 bootstrap realizations using the same local-normalization and maximum-slope procedure employed for the reported SR values. The solid vertical line indicates the reported fitted SR, while the dashed vertical lines indicate the 2.5th and 97.5th percentiles defining the 95% fitting-derived CI.

Table S2. Fitted loaded *Q* and SR with 95% conditional residual-bootstrap CIs for *Dev A–F*.

**Note:** The CIs correspond to the 2.5th–97.5th percentiles obtained from 5,000 conditional residual-bootstrap realizations for each device. They represent fitting-derived uncertainty conditional on the selected weighted Fano–Lorentz line-shape and background model and should not be interpreted as repeated-device experimental CIs.

To quantify the uncertainty associated with the nonlinear line-shape fitting and finite wavelength sampling, a conditional residual-bootstrap analysis was performed for all six devices. For each device, the residuals between the measured high-resolution spectrum and the selected weighted Fano–Lorentz fitted line shape were centered and randomly resampled with replacement to generate 5,000 bootstrap spectra. The same fitting sequence was applied to *Dev A*–*F*. For each bootstrap realization, the resonance wavelength $\lambda_{0}$and linewidth parameter *γ* were re-estimated while retaining the selected fitted line-shape and background parameters. The loaded quality factor was then calculated as *Q* = *λ*_0_/(2*γ*).

The slope rate was independently recalculated for every bootstrap-fitted spectrum using the same procedure as that used for the reported experimental values. Specifically, the fitted transmission line shape was normalized to its local maximum, the maximum absolute derivative was identified, and the corresponding value was converted to dB/nm. The 95% conditional fitting-derived confidence intervals (CIs) of *Q* and SR were defined by the 2.5th and 97.5th percentiles of their bootstrap distributions. All 5,000 bootstrap realizations were successfully fitted for each device, and none reached the imposed lower bound of *γ*.

Table S2 summarizes the fitted values and the corresponding 95% CIs. The complete bootstrap distributions are shown in Figs. S4 and S5 for *Q* and SR, respectively. *Dev A* and *B* exhibit relatively narrow distributions, while the uncertainty progressively increases for spectra that are less strongly constrained by the finite sampling. In particular, *Dev D* exhibits the broadest distribution, consistent with the sensitivity of its very narrow fitted resonance to the wavelength sampling interval. Nevertheless, the bootstrap distributions retain a dominant concentration around the reported fitted values. These intervals therefore provide a quantitative estimate of the fitting sensitivity of *Q* and SR, rather than repeated-device or fabrication-related statistics.

Table S3 Comparison of reported passive or quasi-passive Fano/asymmetric ring-resonator platforms in terms of operating wavelength, Q factor, ER, and SR.

Table S3 summarizes representative passive or quasi-passive integrated Fano/asymmetric microring-resonator platforms reported in the literature [1], [2], [3], [4], [5], [6], including their operating wavelength, *Q* factor, ER, and SR, together with the values obtained in this work. The comparison is intended to provide a representative device-level benchmark rather than a strictly normalized one-to-one comparison, since the reported devices differ in resonance architecture, asymmetry-generation mechanism, operating wavelength, and SR extraction method. Nevertheless, the surveyed studies consistently show that engineered asymmetric resonances are primarily exploited to enhance the usable spectral slope for sensing and signal transduction, as demonstrated in silicon MRR-coupled interferometric structures, hole-perturbed LNOI microrings, and compact Si_3_N_4_ Fano platforms. In this context, the present Ge-OI device operating at 4.2 μm highlights that a competitive SR can be achieved in the mid-infrared through local phase perturbation and asymmetric line-shape engineering, while maintaining a high Q factor.

**References**

[1]	W. Zhang, W. Li, and J. Yao, “Optically tunable Fano resonance in a grating-based Fabry–Perot cavity-coupled microring resonator on a silicon chip,” *Opt. Lett.*, vol. 41, no. 11, p. 2474, Jun. 2016, doi: 10.1364/OL.41.002474.

[2]	C. Zhang *et al.*, “Photonic thermometer with a sub-millikelvin resolution and broad temperature range by waveguide-microring Fano resonance,” *Opt. Express*, vol. 28, no. 9, p. 12599, Apr. 2020, doi: 10.1364/OE.390966.

[3]	L. Gu *et al.*, “Fano resonance lineshapes in a waveguide-microring structure enabled by an air-hole,” *APL Photonics*, vol. 5, no. 1, p. 016108, Jan. 2020, doi: 10.1063/1.5124092.

[4]	Y. Yuan, R. Zhang, Y. He, Y. Zhang, and Y. Su, “Fano Resonance with Sharp Transmittance Slope Based on High-Q Factor Multi-Mode Micro-Ring Resonator,” in *Asia Communications and Photonics Conference 2021*, Shanghai: Optica Publishing Group, 2021, p. W2G.3. doi: 10.1364/ACPC.2021.W2G.3.

[5]	Z. Zhang *et al.*, “Fano-resonant electric field sensor based on the thin film lithium niobate micro-ring with enhanced sensitivity,” *Opt. Express*, vol. 33, no. 12, p. 25210, Jun. 2025, doi: 10.1364/OE.565105.

[6]	J. H. Mendoza-Castro *et al.*, “Engineered fano resonances in a compact Si3N4 photonic crystal nanobeam-microring platform for multi-cladding environments,” *Sci. Rep.*, vol. 16, no. 1, p. 7347, Feb. 2026, doi: 10.1038/s41598-026-35490-w.
